# Variation in Lateral Plate Quality in Threespine Stickleback from Fresh, Brackish and Marine Water: A Micro-Computed Tomography Study

**DOI:** 10.1371/journal.pone.0164578

**Published:** 2016-10-20

**Authors:** Elisabeth Wiig, Janne E. Reseland, Kjartan Østbye, Håvard J. Haugen, Leif A. Vøllestad

**Affiliations:** 1 Department of Biosciences, Centre for Ecological and Evolutionary Synthesis (CEES), University of Oslo, PO Box 1066, Blindern, Oslo, N-0316, Norway; 2 Department of Biomaterials, Faculty of Dentistry, University of Oslo, PO Box 1109, Blindern, Oslo, N-0317, Norway; 3 Department of Forestry and Wildlife Management, Hedmark University College, Campus Evenstad, Elverum, NO-2418, Norway; Federal University of Rio de Janeiro, BRAZIL

## Abstract

**Introduction:**

It is important to understand the drivers leading to adaptive phenotypic diversity within and among species. The threespine stickleback (*Gasterosteus aculeatus*) has become a model system for investigating the genetic and phenotypic responses during repeated colonization of fresh waters from the original marine habitat. During the freshwater colonization process there has been a recurrent and parallel reduction in the number of lateral bone plates, making it a suitable system for studying adaptability and parallel evolution.

**Objective:**

The aim of this study was to investigate an alternative evolutionary path of lateral plate reduction, where lateral plates are reduced in size rather than number.

**Materials and Methods:**

A total of 72 threespine stickleback individuals from freshwater (n = 54), brackish water (n = 27) and marine water (n = 9) were analysed using microcomputed tomography (μCT) to determine variation in size, thickness and structure of the lateral plates. Furthermore, whole-body bone volume, and bone volume, bone surface and porosity of lateral plate number 4 were quantified in all specimens from each environment.

**Results:**

The results showed a significant difference in plate size (area and volume) among populations, where threespine stickleback from polymorphic freshwater and brackish water populations displayed lateral plates reduced in size (area and volume) compared to marine stickleback

**Conclusions:**

Reduction of lateral plates in threespine stickleback in fresh and brackish water occurs by both plate loss and reduction in plate size (area and volume).

## Introduction

A core assignment in evolutionary biology is to understand the drivers leading to adaptive phenotypic diversity within and among species. A particularly useful way of investigating such diversity is to study instances of parallel evolution or adaptive radiations [1,2]. The threespine stickleback (*Gasterosteus aculeatus*), a teleost fish common throughout the northern hemisphere, provides a remarkable case of parallelism in adaptive traits [3]. Following the end of the last glacial period 10 000 years ago, marine threespine stickleback colonized numerous freshwater habitats that became available as the Pleistocene ice sheets retreated. Threespine stickleback in freshwater and brackish water environments diverged phenotypically from their marine ancestor and show extensive variation in morphology, behaviour and diet [4,5]. It has therefore become a model organism in evolutionary biology.

**Fig 1:**
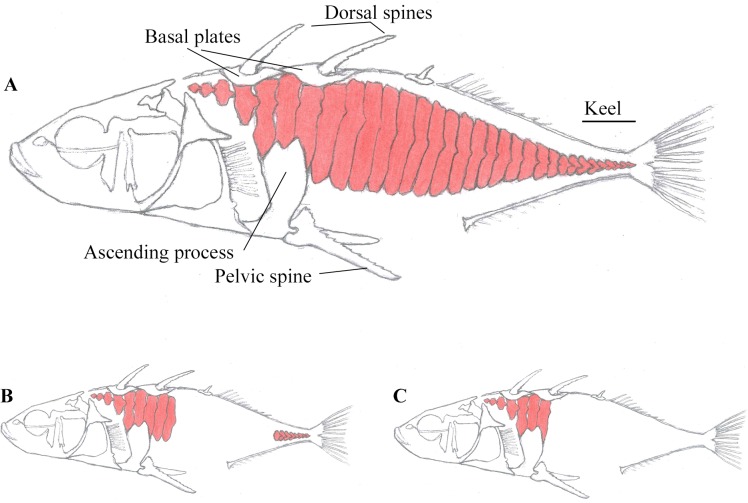
Distribution and variation of lateral plates in threespine stickleback. The complete plate morph (A) with up to 35 lateral plates on each side, covering the anterior region until the caudal peduncle, the partial plate morph (B) lack one or more plates in the midsection or in the keel, and the low plate morph (C) with only plates in the anterior region.

A prominent feature of the threespine stickleback is its armour of up to 35 lateral bone plates on each side of the body [3–6]. In addition to lateral plates, the threespine stickleback is equipped with three dorsal and one pair of pelvic spines, which lock in an erect position if attacked [7]. While marine threespine sticklebacks have a complete row of lateral plates (see Fig 1A), populations in freshwater and brackish water typically display reduced armour with fewer lateral plates [6,8–10]. Variation in number of lateral plates is categorized as plate morphs; the complete plate morph (Fig 1A), the partial plate morph (Fig 1B) typically missing plates in the middle region and the low plate morph (Fig 1C) with only plates in the anterior region [11]. Rarely, plateless stickleback and stickleback with reduced dorsal and/or pelvic girdle also occur [3,10,12,13].

The development of the lateral plates is strongly influenced by several genes, in particular the *ectodysplasin* (*Eda*) gene that to a large degree is controlling the number of armour plates [14,15]. A recessive allele of the *Eda* gene is associated with the reduced plate morphs, where homozygous individuals (aa) are most often low plated, heterozygous individuals (Aa) typically are partial plated, and the completely plated morph is most often homozygous (AA) [15,16]. The extensive repeated occurrences of the low-plated *Eda* genotype in freshwater populations suggest that reduction of lateral plates is under selection, either directly or indirectly [15,17]. Several hypotheses have been proposed for lateral plate reduction in freshwater environments including shifts in predation pressures[18–20], swimming and buoyancy [21–23] and differences in osmoregulation [5,24]. Another hypothesis suggests that plate reduction seen in freshwater is due to limited availability of minerals essential for bone development [10] and a preferred allocation of energy to growth and reproduction instead of armour plates [25].

In a few freshwater lakes, sticklebacks with only the complete plate morph are found; i.e. they are monomorphic and likely genetically fixed for the complete plate genotype of the *Eda* gene. If the development of lateral plates is costly, it is probable that natural selection has found other ways to reduce lateral plate investment. One such alternative way to lateral plate reduction could be reduction in the size of lateral plates. A small-plate phenotype was suggested by Leinonen et al. as an alternative to plate loss for freshwater populations holding only complete plate morph [24,26,27]. Alternatively, a reduced investment into the lateral plates can be accomplished by reducing the quality of the bone structure. The microstructure of lateral plates has earlier been investigated by Song et al. and Lees et al., however these studies included only marine threespine stickleback [28,29].

Despite extensive research on lateral plate reduction in freshwater stickleback, we still know little regarding variation in plate volume, area and porosity across the range of environments that threespine stickleback inhabit. Accordingly, the aim of this study was to investigate variation in area, volume and porosity of lateral plates in threespine stickleback from a range of habitats (ancestral marine, brackish water and freshwater). Here, we study variation in area, volume and porosity in lateral plates of complete plate individuals from these different environments, as complete plate morph in freshwater are expected to have developed reduced armour compared to brackish and marine stickleback. Secondly, we compared area, volume and porosity in lateral plates in the three plate morphs in one brackish water locality and in one freshwater site.

## Materials and Methods

### Samples

Threespine sticklebacks were sampled during spring and summer of 2011 and 2012. Two freshwater (FW) lakes (Gardatjørna—60°17′41.10″ N, 05°05′09.81″ E; and Asdøltjern—59°52′25.92″ N, 10°19′44.10″ E) and one brackish water (BW) lagoon (Engervann– 59°53′43.29″ N, 10°31′50.70″ E, conductivity: 60–3000 mSm^-1^) were selected for sampling [30]. Sampling was done by hand-held dip nets and baited plexiglas traps [31]. Collected stickleback were immediately euthanized with a lethal dose of benzocaine and stored in 96% ethanol. A fishing permit was granted by Norwegian Directorate for Nature Management, and care was taken to minimize suffering of the fish. A marine sample from the Barents Sea (M) (72°24′32.88″-75°26′54.00″ N, 30°18′52.96″-31°23′44.86″ E) was acquired with the assistance of the Institute of Marine Research (Bergen, Norway). Here, the threespine stickleback were sorted out from the by-catch of trawl catches taken at 255–387 meters depth during January-March 2012, frozen and later stored in ethanol, before being transferred to the University of Oslo.

The Barents Sea and the Gardatjørna samples consisted solely of complete plate morph individuals (Fig 1A). No plate reduction by plate loss has been observed in the Gardatjørna population, it is thus assumed to be a monomorphic population consisting of only complete plate morph individuals. The populations in the lake Asdøltjern and the brackish water Engervann holds all three plate morphs: a complete plate morph, a partial plate morph (Fig 1B) and a low plate morph (Fig 1C). Nine individuals for each plate morph in each population were sampled, in total 72 threespine stickleback.

### Measurement and selection criteria

Total length, defined as length from the tip of snout to the end of the caudal fin, in natural position, was measured for each specimen. All individuals used were larger than a minimum length of 40 mm to ensure that the bone plate development in all individuals was completed [5,14,32].

Injured and heavily parasitized individuals were excluded. Several parasites that infect three-spine stickleback, such as *Schistocephalus sp*., alter natural foraging and behaviour [33], thus such parasites may putatively affect normal bone development. Further, investment into reproduction, in particular female investment into eggs, may also affect investment in developing bone armour structures. Males were selected to exclude such investment. However, the Barents Sea sample did not provide enough males, thus five non-gravid females were included. Sex identification was done by genotyping or, in few specimens by distinguishing male colour display and visual examination of the gonads.

Pectoral fins and caudal fin were collected for potential molecular sex determination and stored in 96% ethanol (EtOH). DNA was extracted using a salt-extraction method [34] and amplified with primers for *Idh* (isocitrate dehydrogenase), F: 5’GGGACGAGCAAGATTTATTG 3’ and R: 5’TTATCGTTAGCCAGGAGATGG3’ designed by Peichel et al. [35]. The PCR cycles were as following: 95°C for 1 minute and 45 sec, 56°C for 45 sec, 72°C for 45 sec, 5 cycles of: 94°C for 45 sec, 56°C for 45 sec, 72°C for 45 sec, 30 cycles of 90°C for 45 sec, 56°C for 45 sec, 72°C for 45 sec followed by 72°C for 10 minutes and then cooled down to 6°C. PCR products were visualized on an ethidium bromide stained 2% agarose gel in TAE buffer, with males identified as heterogametic markers with band at 302 bp and 271 bp, and females as homogametic marker with band at 302 bp.

### Micro-computed tomography

Three-dimensional description of bone structures was accomplished by the use of micro-computed tomography (μCT). For each individual the entire fish was scanned and reconstructed providing a whole body reconstructed model. Samples were scanned with SkyScan 1172 desktop μCT (Bruker, Kontich, Belgium), with a source voltage of 59 kV and with 167 μA source current and no filter. Samples were scanned with 15 μm voxel size. Reconstruction was performed by the use of NRecon, version 1.6.4.8 (Bruker, Konitich, Belgium). When reconstructing, the model was corrected for beam-hardening (15%) [36].

Variation in investment into lateral plates among individuals was compared by isolating one particular lateral plate (lateral plate number 4; LP4) (Fig 1A) for each specimen, this was done by manually drawing the region of interest around LP 4, excluding all other bone structures, using CT-Analyzer, version 1.13.4.0 (Bruker, Konitich, Belgium). This lateral plate is positioned under the first basal plate, between cleithrum and ascending process. LP4 was present in all individuals, however some individuals were missing the first three lateral plates positioned before LP4, here the lateral plate in the LP4 position was identified as LP4, even if it was the first lateral plate in the set. Structural parameters such as bone volume (mm^3^), bone surface area (mm^2^), total porosity (%, percentage of volume of open and closed pores in the isolated lateral plate) of the isolated lateral plate, and total bone volume of the whole specimen were abstracted from the data set using the same CTanalyzer software. Visual 3D description and comparison of the whole body was achieved with μCT visualisation software CTvox version 2.5.0.0 (Bruker, Konitich, Belgium). This software was also used for counting lateral plates on both sides of the fish.

### Statistical analysis

We tested for differences in various phenotypic traits among population and morph using generalized or general linear models (GLM). First, we compared the complete plate morph individuals from the four different populations. The total number of plates was compared using a GLM with a Poisson error distribution, population as a fixed factor and fish length as covariate. For all other tests, with total plate volume, and area, volume and porosity of lateral plate no 4 (LP4), we used a GLM with normal error distribution and fish length as covariate.

Secondly, we compared the three different morphs occurring in sympatry in the Engervann and Asdøltjern populations. Here, we tested for variation in total plate volume, and area, volume and porosity of LP4 among morphs and between populations. Also here we used a GLM with normal error distribution and fish length as covariate.

The various glm-models were then evaluated further using the Tukey HSD (post hoc) test to test for differences among populations or morphs. The response variables total bone volume; LP4 bone volume, area and porosity as well as fish length were log-transformed to normalize residuals. These transformations were appropriate and produces normally distributed model residuals. All statistical analyses were performed using JMP 12.0.1 (www.jmp.com).

## Results

### Comparison of the complete plate morph among marine, fresh- and brackish water

**Fig 2:**
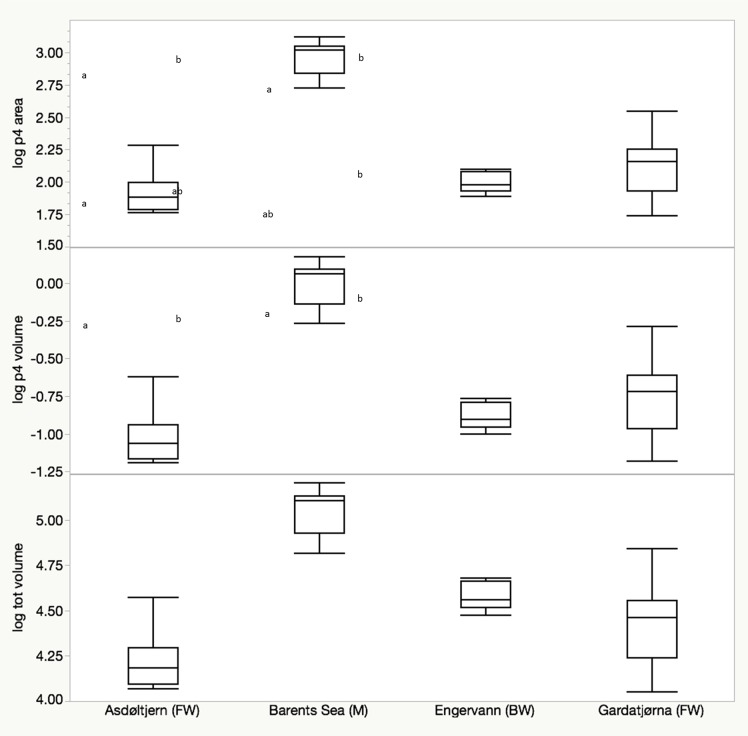
Among-population variation in lateral plate quality among complete plate stickleback. Box-plot of log-transformed predicted trait values (total lateral plate volume (mm^3^), volume (mm^3^) and area (mm^2^) of lateral plate no 4) from Asdøltjern (FW), Barents Sea (M), Engervann (BW) and Gardatjørna (FW). Different letters indicate significant differences based on Tukey HSD tests.

Mean total number of lateral plates on both sides in complete plate morphs from all four populations differed between 62.6 and 63.8 plates (Table 1), however plate number did not differ among the four populations (generalized linear model, Poisson distribution; χ^2^ = 0.12, df = 3, P = 0.989). Total bone volume (mm^3^) was as expected significantly correlated with fish length (F_1, 31_ = 25.12, P < 0.001) and differed significantly among populations (F_3, 31_ = 3.61, P = 0.024) (Fig 2). In general, the length-adjusted total bone volume was smaller for the Asdøltjern (FW) and Engervann (BW) fish than for the Gardatjørna (FW) and Barents Sea fish (M) (Fig 2).

**Table 1:** Quality and size of lateral plates in threespine stickleback from marine (M), brackish water (BW) and freshwater (FW).

Location	Morph	Length (cm)	Total plate no.	Bone volume (total) mm^3^	Bone volume (plate 4) mm^3^	Bone surface area(plate 4) mm^2^	Total porosity (plate 4) %
Barents Sea(M)	Complete	6.2±0.4	63.8±1.2	162.7±46.9	1.029±0.254	19.57±2.69	83.3±8.4
Engervann (BW)	Complete	5.2±0.2	62.9±1.5	98.6±17.0	0.421±0.085	7.46±1.27	73.2±7.0
	Partial	5.2±0.3	42.4±7.1	76.6±17.9	0.327±0.099	6.39±1.62	76.7±3.3
	Low	5.0±0.4	14.3±3.4	64.5±12.8	0.263±0.086	5.26±1.87	68.4±6.8
Asdøltjern (FW)	Complete	4.9±0.4	62.6±1.7	70.1±20.1	0.372±0.101	7.00±1.62	70.9±3.6
	Partial	5.3±0.4	44.6±11.5	75.4±28.7	0.359±0.133	6.85±2.69	70.4±3.0
	Low	5.1±0.4	13.2±1–6	63.3±13.3	0.298±0.066	5.65±1.17	69.0±2.9
Gardatjørna (FW)	Complete	4.9±0.5	63.3±0.9	85.8±14.1	0.491±0.135	8.77±2.62	70.8±5.6

Data presented as means ± sd for various phenotypic traits

There was a significant difference in bone volume (mm^3^) and surface area (mm^2^) of the isolated lateral plate 4 among populations (volume: F_3, 31_ = 10.50, P < 0.001; area: F_3, 31_ = 16.72, P < 0.001) after adjusting for fish length (volume: F_1, 31_ = 32.40, P < 0.001; area: F_1, 31_ = 36.24, P < 0.001) (Fig 2). The Barents Sea and Gardatjørna fish had larger LP 4 (area and volume) than the Asdøltjern and Engervann fish. There was no difference in LP4 porosity among the populations (F_3, 31_ = 2.46, P = 0.081).

**Fig 3:**
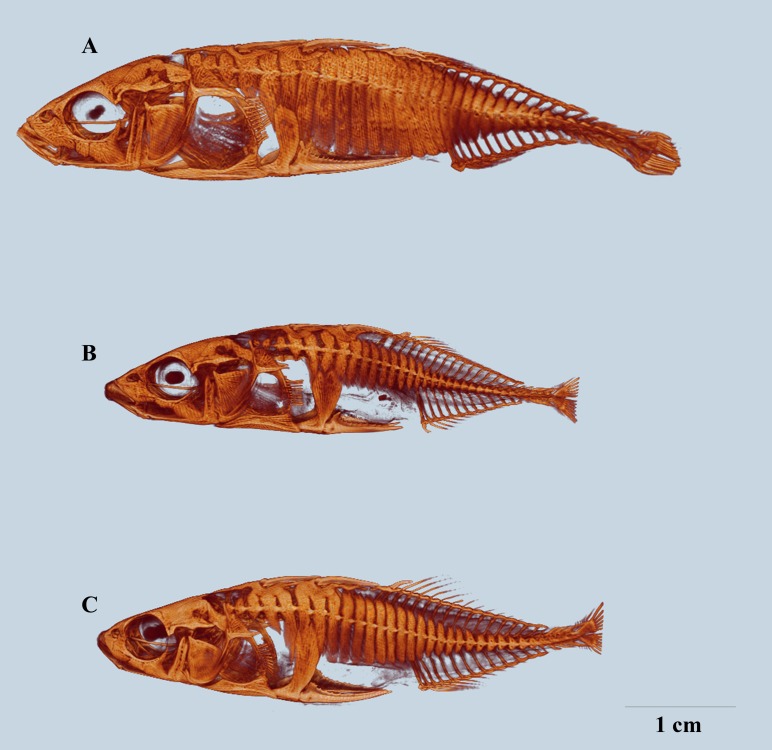
The Barents Sea fish (A) display a complete and rigid armour; lateral plates are covering the majority of the fish body and abutting. Complete plate morph from Asdøltjern (B), displaying reduction in size of lateral plates. Complete plate morph from Gardatjørna (C), displaying reduction in size of lateral plates to lesser extent. Representative images for the populations. Scale bar 1 cm.

**Fig 4:**
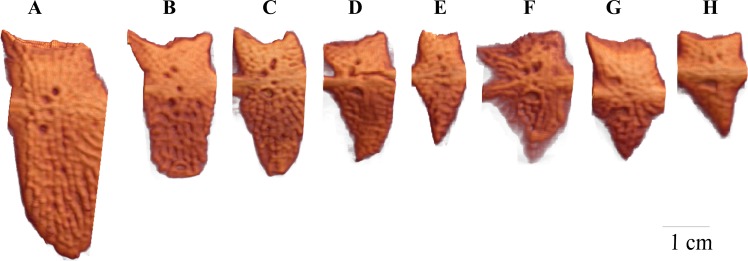
Representative images for the populations of the isolated LP 4 from the Barents Sea (A), Gardatjørna (B), from Engervann; complete plate morph (C), partial plate morph (D), low plate morph (E) and from Asdøltjern; complete plate morph (F), partial plate morph (G) and low plate morph (H). Lateral plate size is not corrected for fish length.

By visual comparison of reconstructed μCT models of whole fish, it was evident that lateral plate size varied with the plate position and among populations. Additionally, lateral plates varied in shape and the degree to which they overlapped with other bone structures (Fig 3, Fig 4). The Barents Sea fish (Fig 3A, Fig 4A) displayed heavy armour. The lateral plates in the anterior region were of oval to rectangular shape and overlapping with the first and second basal plate and the ascending process of the pelvic girdle, lateral plates at the midsection were high and rectangular in shape and abutting. The lateral plates at the keel were smaller compared to the other lateral plates and triangular in shape with the bony protrusion enlarged so that it formed a bony bridge with the next lateral plate. In comparison to the Barents Sea fish, complete plate morph individuals from Asdøltjern (Fig 3B, Fig 4F) displayed reduced armour and the lateral plates in the anterior region were small, round and spaced. The overlap with the basal plates or the ascending process was minimal. Lateral plates at the midsection were narrow and short with minimal abutting. Lateral plates at the keel were similar to the Barents Sea fish, with some reduced bony protrusion. Gardatjørna fish (Fig 3C, Fig4 B) displayed a less reduced plate size in the anterior region and at the keel, compared to Asdøltjern fish. Lateral plates in the midsection were gradually reduced in size posteriorly with small lateral plates before the keel.

### Comparison among plate morphs in fresh- and brackish water

**Fig 5:**
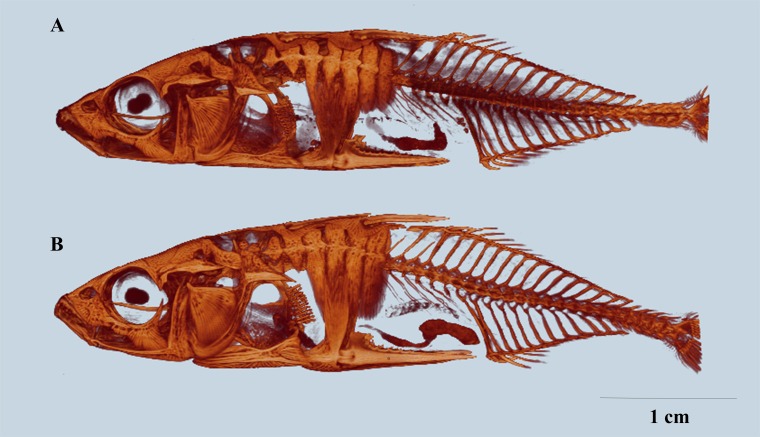
Partial plate morph morph from population Asdøltjern (A) displaying lateral plates in the anterior region that appear reduced in height and width, in addition to loss of plates in the midsection and missing LP 1. Low plate morph from population Engervann (B) with only plates in the anterior region that appear reduced in height. Representative images for the populations are presented.

The complete-plated individuals in the brackish water population in Engervann and in the freshwater population in Asdøltjern were similar (Tables 2 and 3). These populations also harboured partial and low plated individuals (Fig 5). Plate number of the partial morph differed between 42.4 and 44.6 plates, and for the low plated morph between 13.2 and 14.3 plates.

**Table 2:** Summary statistics for test for differences in trait values for low, partial and complete plated stickleback morphs from Asdøltjern and Engervann.

Parameters	Bone volume (plate 4) mm^3^	Bone volume (total) mm^3^	Bone surface area(plate 4) mm^2^	Total porosity(plate 4) %
Location	F_1, 49_ = 0.39, P = 0.535	F_1, 49_ = 7.39, P = 0.009	F_1, 49_ = 0.42, P = 0.518	F_1, 49_ = 3.02, P = 0.089
Morph	F_2, 49_ = 8.81, P<0.001	F_2, 49_ = 11.14, P<0.001	F_2, 49_ = 7.61, P = 0.001	F_2, 49_ = 4.03, P = 0.024
Length (log-transformed)	F_1, 49_ = 17.62, P<0.001	F_1, 49_ = 62.33, P<0.001	F_1, 49_ = 20.01, P<0.001	F_1, 49_ = 4.46, P = 0.040

Summary statistics include F- and p-values. All trait values are log-transformed, and adjusted for size and location effects.

**Table 3:** Trait values in low, partial and complete plate morph from Asdøltjern and Engervann.

Morph	Bone volume (plate 4) mm^3^	Bone volume (total) mm^3^	Bone surface area(plate 4) mm^2^	Total porosity(plate 4) %
Low	-1.292±0.062 B	4.162±0.040 B	1.674±0.055 B	4.230±0.015 B
Partial	-1.161±0.062 B	4.245±0.040 B	1.814±0.055 AB	4.291±0.016 A
Complete	-0.934±0.061 A	4.421±0.040 A	1.976±0.055 A	4.277±0.016 AB

Trait values (± se) adjusted for length. Values with same letters are not significantly different based on Tukey HSD test

Total bone volume differed significantly between populations and among plate morphs (Table 2). Total bone volume was generally larger in Engervann fish than in Asdøltjern fish, and the complete plate morph had larger total bone volume than the low and partial plated morph. For plate 4, volume and area did not differ between populations, but complete plate morph individuals had larger LP 4 than both low and partial plated individuals (Fig 4C–4H). There was no significant difference in Plate 4 porosity between the low and partial plated individuals, and there was no difference in plate porosity between populations (Table 3).

## Discussion

This study revealed significant variation in size and quality of the lateral plates of threespine stickleback from populations in different environments. Stickleback from fresh and brackish water show lateral plates reduced in size relative to the marine fish. The Barents Sea fish displayed a robust armour with large (area and volume) lateral plates that were partly overlapping. However, the fish from the freshwater lake Gardatjørna also had large (area and volume) plates; thus salinity itself does not appear to be the sole causation of the reduction in plate size.

Leinonen et al. hypothesized that individuals with complete plates in fresh water are subject to reduction in size of the plates, as an alternative way to reduce their armour, whereas populations with sufficient frequency of the recessive low plate *Eda* allele would not reduce their plate size [26]. However, we found a reduction in area and volume of lateral plate in individuals of all morphs from the fresh and brackish waters studied. Although reduction was observed in all morphs from fresh and brackish water, the reduction in area and volume of LP 4 was significant for the two polymorphic populations in this study. The Gardatjørna population with only complete plate morphs is assumed to be fixed for the complete plate *Eda* allele. If there is strong selection for reduced investment into lateral plates in fresh water, a strong and directed selection for smaller plates would be expected, such as the small-plate phenotype described by Leinonen et al., as there are no other paths for reduction [26,27]. Yet, we observed that the stickleback in Asdøltjern, a freshwater lake holding all three morphs had smaller lateral plates than the monomorphic population in the Gardatjørna. Our findings are thus different from previous observation of generally smaller plates in threespine stickleback in freshwater, and in particular in morphs homozygous for the *Eda* complete plate allele [26]. However, Gardatjørna fish had noticeably reduced size in lateral plates in the middle section, these plates were not individually isolated and measured like LP 4, but could be observed in the reconstructed model from the ct scan. This observed reduction in size of lateral plates at the midsection of Gardatjørna fish could be connected to the development of lateral plates.

Lateral plates start to develop when the fish is around 12 mm long, 4–5 weeks after hatching [37–39]. The development of the lateral plates is a long and complex process, where lateral plates are developed and ossified in a particular order [9,32]. The lateral plates in the anterior region (LP 3-LP 8) form overlapping structures with the basal plates and the ascending process and together they constitute a rigid belt-like structure [6]. They are considered to constitute an important functional structure for erection of the dorsal and pelvic spines when attacked by predators. The isolated lateral plate in this study; LP 4, is perhaps less likely to be reduced in size in response to environment due to its importance as a functional structure and early development. Since this study only comprises one complete plate only fresh water population, this will only be speculative until future research.

Studies by Colosimo et al. and Indjeian et al. have identified genes controlling lateral plate height and width, and several of these genes are located the chromosome region associated with plate morph [15,40]. We did observe significant difference in LP 4 size (area and volume), with population Asdøltjern and population Engervann having smaller LP 4 than marine stickleback and stickleback from the momomorphic population Gardatjørna. Further, when testing between morphs in population Asdøltjern and Engervann there was no significant difference in LP 4 size (area and volume) between low plate morph and partial plate morph. However, the complete plate morph had larger LP 4 (area and volume) than the partial and low plate morph. If the lateral plate size responded independently to some environmental condition in fresh water, we would expect a greater reduction in plate size in the complete plate morph than in the two other morphs as a cost of producing a large number of plates. Our results may suggest a genetic linkage between reduction in plate size and plate loss. Further study of lateral plate size should include more populations and may avail in combing the use μCT with genetic and genomic studies.

Albeit total bone volume was larger in population Engervann than the fresh water population Asdøltjern, the two populations appear similar across morphs. Brackish water systems are strongly connected to both marine and freshwater habitats, and gene flow among habitats and populations may occur. Population Engervann might comprise some freshwater stickleback that have dispersed from upstream freshwater locations in addition to stickleback from the marine environment. The strong similarity between the brackish and freshwater populations may indicate that this particular brackish water population could be influenced by past or present gene flow from fresh water populations. Alternatively, the three lateral plate morphs could have originated in the brackish water environment itself, where the phenotypic similarity between Asdøltjern and Engervann is due to similar selection pressures as Engervann is highly influenced by freshwater inputs.

Finally, in comparison to the detailed studies of lateral plates by Song et al. and Lees et al. which revealed sandwich-like structures and surface topography of rows of ridges with nodules [28,29], we observed similar surface topography by μCT, with little variation between morphs and population (data not shown). The size and volumes of lateral plates in marine stickleback were similar to the results presented by Song et al. [28].

## Conclusion

We conclude that threespine stickleback in fresh and brackish water may reduce lateral plates in various ways; by reducing reducing number of plates and by reducing plate size. Further, the alternative path by reducing plate size is not restricted to complete plate morph, some stickleback reduce size of lateral plates in addition to reducing number.

## Supporting Information

S1 Table (XLSX)
